# Optimizing human activity patterns using global sensitivity analysis

**DOI:** 10.1007/s10588-013-9171-0

**Published:** 2013-12-10

**Authors:** Geoffrey Fairchild, Kyle S. Hickmann, Susan M. Mniszewski, Sara Y. Del Valle, James M. Hyman

**Affiliations:** Defense Systems and Analysis Division, Los Alamos National Laboratory, Los Alamos, NM, USA; Department of Mathematics, Center for Computational Science, Tulane University, New Orleans, LA, USA; Computer, Computational, and Statistical Sciences Division, Los Alamos National Laboratory, Los Alamos, NM, USA; Defense Systems and Analysis Division, Los Alamos National Laboratory, Los Alamos, NM, USA; Department of Mathematics, Center for Computational Science, Tulane University, New Orleans, LA, USA

**Keywords:** Global optimization, Global sensitivity analysis, Sample entropy, Agent-based modeling, Bayesian Gaussian process regression, Harmony search

## Abstract

Implementing realistic activity patterns for a population is crucial for modeling, for example, disease spread, supply and demand, and disaster response. Using the dynamic activity simulation engine, DASim, we generate schedules for a population that capture regular (e.g., working, eating, and sleeping) and irregular activities (e.g., shopping or going to the doctor). We use the sample entropy (SampEn) statistic to quantify a schedule’s regularity for a population. We show how to tune an activity’s regularity by adjusting SampEn, thereby making it possible to realistically design activities when creating a schedule. The tuning process sets up a computationally intractable high-dimensional optimization problem. To reduce the computational demand, we use Bayesian Gaussian process regression to compute global sensitivity indices and identify the parameters that have the greatest effect on the variance of SampEn. We use the harmony search (HS) global optimization algorithm to locate global optima. Our results show that HS combined with global sensitivity analysis can efficiently tune the SampEn statistic with few search iterations. We demonstrate how global sensitivity analysis can guide statistical emulation and global optimization algorithms to efficiently tune activities and generate realistic activity patterns. Though our tuning methods are applied to dynamic activity schedule generation, they are general and represent a significant step in the direction of automated tuning and optimization of high-dimensional computer simulations.

## 1 Introduction

High-dimensional computer models for simulating real world phenomena have many variables and present a difficult challenge in understanding the relationship between input and output. Known as the *curse of dimensionality*, a full space analysis of the nature of input-output relationships is NP-complete, scaling exponentially as *s^n^*, where *s* is the number of sample values for each of the *n* input variables (Rabitz and Aliș 1999). This paper presents an efficient method for determining these input-output relationships in high-dimensional models using a combination of global optimization and global sensitivity analysis. We demonstrate our method using a model of human activity and movement.

Human activity and movement patterns are complex and notoriously difficult to model (Berry et al. 2002). Large variations in movement patterns stem from demographic, geographic, and temporal differences. Quantifying the effects of these differences on human activity/schedules provides a difficult but important challenge (González et al. 2008). Realistic human activity and movement models are fundamental components for agent-based infrastructure simulations. These models use human activity patterns to simulate complex systems including epidemics (Eubank et al. 2004; Colizza et al. 2007; Mniszewski et al. 2008; Stroud et al. 2007), traffic (Kitamura et al. 2000), and natural disaster response (Pan et al. 2007). Despite their importance, models typically simplify the complexity of human movement and rely on estimates such as static activity patterns. The static approach results in a *Ground-hog* *Day*-like effect, where every person performs the same activities day in and day out according to a fixed schedule. Since the schedule cannot be modified based on exogenous events, the schedule will inevitably repeat over some finite time scale.

The level of realism required in a model of natural phenomenon depends upon the scenario being modeled and the questions being addressed (Burton 2003; Burton and Obel 1995). In epidemic modeling, capturing emergent human behavior is crucial for accurately forecasting the spread of disease and the impact of mitigation strategies. Similarly, for modeling disaster response during a natural or man-made event, understanding people’s activities before and after the event will help emergency responders allocate resources. Finally, supply and demand modeling of various utilities (e.g., water, electricity, and communications) depends on the population’s activities as they move throughout the day. Therefore, capturing realistic activity patterns can help improve modeling efforts and save lives during emergencies.

We have built on the previous body of activity pattern research. Germann et al. presented a study analyzing mitigations for a pandemic influenza in the United States (Germann et al. 2006). In their study, 12-hour schedules were cycled to direct the activities of seven different mixing groups consisting of work, school, day care, play group, neighborhood, neighborhood clusters, and communities. Paleshi et al. performed a similar study that featured stricter mixing patterns according to four coarsely-defined demographic groups: preschool children (ages <1–4), school children (5–18), adults (19–64), and seniors (≥65) (Paleshi et al. 2011). In Stroud et al. (2007) and Mniszewski et al. (2008), epidemic simulations rely on static schedules with individuals cycling through nine different activities. Additionally, in contrast to other studies, individuals temporarily deviate from their schedules when ill, and parents stay home with sick children. Weekday schedules were further distinguished from weekends and holidays by replacement of work or school with home for a portion of the population in a study of social contact patterns and their effect on the spread of disease (Del Valle et al. 2008); it was found that the lack of weekday and weekend activities can greatly overestimate the impact of disease spread. Brockmann et al. moved beyond the realm of static-based schedules by considering random walks as a proxy for human movement based on trajectories of almost 500 thousand dollar bills (Brockmann et al. 2006). González et al. studied the paths of 100 thousand mobile phone users and showed that humans do not behave randomly; rather, they follow simple reproducible spatial patterns (González et al. 2008). All of these models neglect basic human characteristics based on desire, need, and importance that can impact and change schedules accordingly (e.g., getting sick may force a person to go home early from work, or car maintenance may preclude shopping). A realistic human activity and movement model needs to dynamically take these basic human traits into account (Macy and Willer 2002).

We use the Dynamic Activity Simulator (DASim), previously known as Activity-Sim (Galli et al. 2009), that incorporates activity utility and priority to develop schedules for a population of individuals. DASim generates schedules that give each individual close to the maximal utility that complements their priorities for activities. This allows one to design population schedules by specifying priorities and utilities of a variety of activities for any number of demographic groups. Moreover, new schedules can be generated dynamically during a simulation.

Once these schedules are dynamically generated, it is not immediately apparent if they are realistic for a population. In actual populations, we expect demand hours (i.e., the total number of people participating in an activity aggregated over one hour) for certain activities, such as grocery shopping or working, to be stable on any given weekday. For recreation activities or hospital visits, we expect daily demand hours to fluctuate, with possibly a more stable amount of demand hours on a monthly or quarterly timescale. In this way, regularity of demand hours can be required in population’s schedules to classify traits of certain activities, thus adding realism to the dynamic schedule generation. We propose to quantify an activity schedule’s regularity using the *sample entropy* (SampEn) statistic (Richman and Moorman 2000). That is, the SampEn of the time series associated with DASim output is used to dynamically adjust schedules to be consistent with regular and irregular activity patterns. By tuning SampEn, one can design schedules comprised of activities that occur with a desired level of regularity.

Tuning the SampEn statistic for a schedule can be posed as a high-dimensional optimization problem. Global sensitivity analysis can be used to reduce the dimensionality of the optimization problem by targeting the input parameters in DASim that control the majority of variation in SampEn. The sensitivity analysis was carried out efficiently through the use of Bayesian Gaussian process regression. Once a low-dimensional set of influential parameters is discovered, a global optimization scheme, *harmony search* (HS) (Geem et al. 2001), is used to tune SampEn and therefore adjust the regularity of activities in a schedule. We demonstrate that reducing the search space for HS to only influential parameters results in a more efficient search.

## 2 Methods

### 2.1 Dynamic activities model

DASim is a dynamic parallel agent-based discrete event movement and activity simulator. DASim requires two components to generate schedules: (1) a population with demographic characteristics, and (2) locations with geographic coordinates. DASim can use any population and location data, but the synthetic population we use is based on U.S. census data^1^ and includes various demographic characteristics such as age, gender, income, and status (e.g., worker, student, and stay home). In addition, each person has a household consistent with the census data. Locations are derived from the Dun & Bradstreet business directory database,^2^ which include addresses and business type. Businesses can be aggregated in a geographic area and may include multiple business types such as a shopping mall. DASim integrates all this information to generate realistic schedules according to the person’s preferences and needs.

Activities are defined based on the scenarios of interest. For example, they can be general (e.g., home, work, school, shop, social recreation), more specific (e.g., sleep, personal care, breakfast, lunch, food shopping, morning work, afternoon work), or mixed. Subsets of activities are stratified based on different demographic characteristics such as age, school and worker status, and/or gender. Some examples include children (0–5 years old), youth (6–18 years old), workers (19–64 years old), and seniors (65+ years old). In DASim, each demographic group is assigned an activity set comprised of various allowed activities as demonstrated in Table 1. Each activity in each set has associated constraints, a utility function, and a priority function. These controls provide the ability to finely tune activities for each population group.

**Fig. 1:**
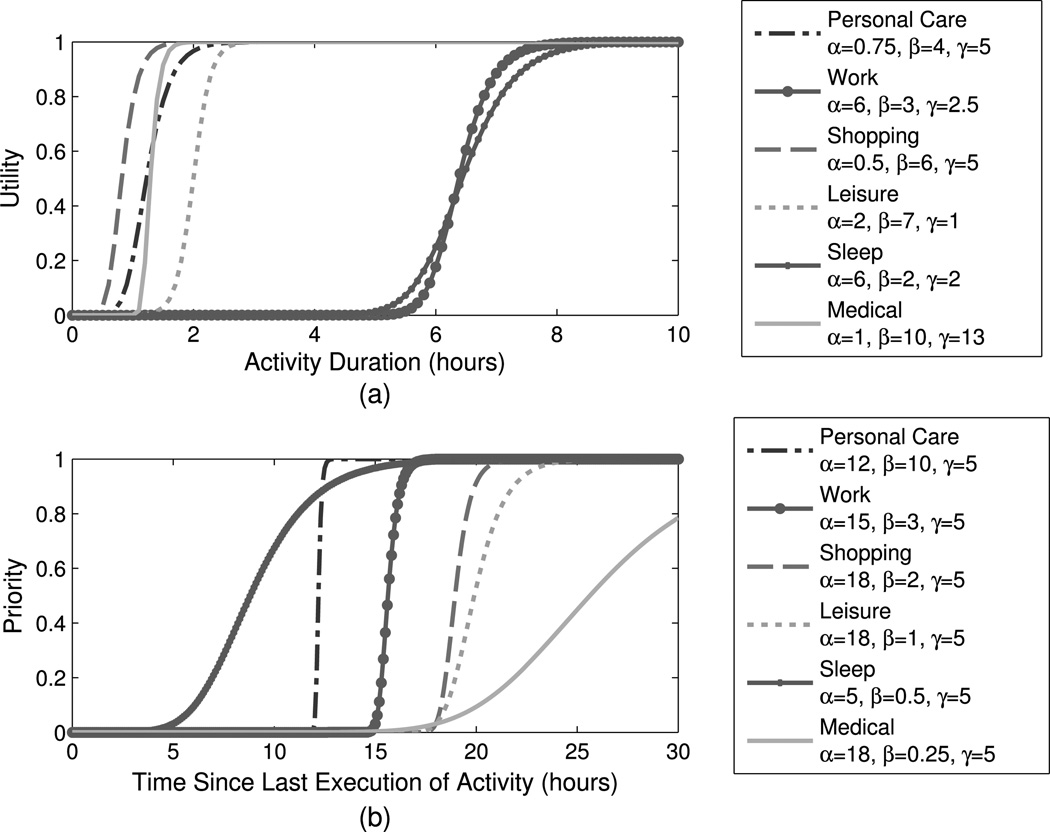
Sample utility and priority functions for a person in the worker demographic group. (**a**) Presents what the worker demographic group’s utility functions might look like. Utility is a function of the duration of an activity. Activity utility is a representation of how desirable or beneficial an activity is. In this example, the maximum utility derived from sleeping is at about 8 hours. This means that sleeping for 8 hours is more beneficial than sleeping for only 6 hours, but there is no benefit to sleeping beyond 8 hours. (**b**) Presents what the worker demographic group’s priority functions might look like. Priority is a function of the time since an activity was last executed. Activity priority is a representation of how important the activity is. In this example, it becomes important for a person to sleep once it has been about 15–20 hours since they last slept. Additionally, a person needs to work once it has been approximately 15 hours since they last worked. The sigmoid function used and graphs shown here are adapted from data presented in Joh et al. (2001)

DASim’s utility and priority functions govern activity benefit and importance, respectively. In practice, utility functions influence activity duration, and priority functions influence the order in which activities occur. Utility increases up to a limiting or maximum useful duration. Priority indicates how often an activity is scheduled given the longest possible time between activity executions. So, the utility is a function of activity duration, *d* ≥ 0, while priority is a function of activity start time, *t* ≥ 0. Utility (*U*) and priority (*P*) functions are represented in DASim by the sigmoid function presented in (Joh et al. 2001),

(1) $$U(d)={\left(1+e^{-\beta_{u}(d-\alpha_{u})}\right)}^{-\gamma_{u}},\;P(t)={\left(1+e^{-\beta_{p}(t-\alpha_{p})}\right)}^{-\gamma_{p}},$$

where α_{*u, p*}_, β_{*u, p*}_, and γ_{*u, p*}_ are activity-specific parameters that determine the function’s offset, slope, and inflection point, respectively. Table 2 describes in more detail how these parameters affect utility and priority. For a more detailed analysis of these parameters and what they mean in practice, the reader is referred to Joh et al. (2001). *U* and *P* vary over the interval [0, 1]. To change a dynamically-generated schedule in DASim, we vary the six parameters (α_*u*_, β_*u*_, γ_*u*_, α_*p*_, β_*p*_, γ_*p*_) for each activity and for each demographic group. Figure 1 demonstrates sample utility and priority functions for several activities using different parameter sets.

A schedule is defined as a set of activities, where each activity has a specified minimum and maximum duration, start and end window, utility and priority functions, maximum travel time, and probability the activity will be performed on a weekend. Activities are scheduled in windows of time (e.g., a 24-hour window means that each person schedules activities 24 hours in advance). A schedule s* is generated by maximizing an objective function that balances the utility of an activity against the priority of all activities and the time it takes to travel to each activity in order to rank schedules,

(2) $$s^{*}=\underset{s\in S}{\text{arg max}}\frac{1}{N}\sum_{i=1}^{N}(U_{a_{i}}(d_{i})-\frac{C}{B}\sum_{r=1}^{B}P_{a_{i}}(t_{r})-\frac{D}{TT_{a_{\text{max}}}}T_{a_{i}}).$$

Here, *s* is a schedule in the set of all possible schedules *S*, *N* is the number of activities in schedule *s*, *U_a_i__* (*d_i_*) is the utility of activity *a_i_* of duration *d_i_*, *C* is the priority multiplier, *B* is the number of all possible activities from which the agent can choose, *P_a_i__* (*t_r_*) is the priority of activity *a_i_* at time *t_r_*, *D* is the travel time multiplier, *T* *T_a_max__* is the maximum travel time for activity *a*, and *T_a_i__* is the travel time for activity *a_i_*.

The two parameters in the objective function, *C* and *D*, weigh the importance of the priority function and travel time constraints, respectively. *C* and *D* are global parameters and apply equally to *all* activities for *all* demographic groups. The three parameters in each of the utility and priority functions are local parameters set on a per-activity basis.

**Fig. 2:**
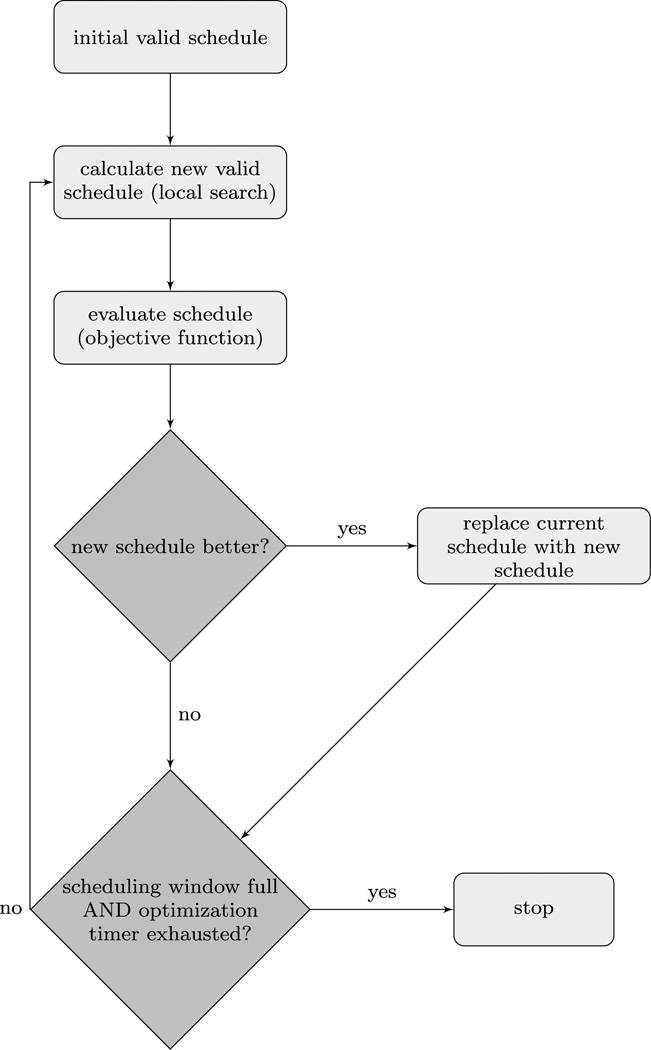
Schematic showing the schedule-designing loop in DASim. The loop begins with a (possibly empty) initial schedule. Using the local search metaheuristic described in the text, a new valid schedule is constructed. The objective function compares the new schedule to the old schedule, and the better schedule is then used. This process continues until the scheduling window is full *and* the optimization timer is exhausted

Schedules are designed using the local search metaheuristic (Lourenço et al. 2003), similar to the method used in Joh et al. (2001). The local search algorithm iteratively adds new activities to a schedule or randomly selects an operator to apply to the schedule from the operators presented in Table 3. Activities are selected randomly from a set specific to each demographic group with probability weighted by priority. Activity duration is chosen using the specified time constraints. Travel distance is calculated relative to the location of the previous activity and is calculated as Euclidean distance, not as road or travel distance. To calculate travel time, we divide the travel distance by the average speed (fixed at 16 m/s). The objective function is used to evaluate proposed schedule changes. The schedule for a time window is complete when full (i.e., when there is no unaccounted-for time in the individual’s scheduling window) and a fixed number of optimization iterations have been completed. In our experiments, we use 10 iterations of the local search algorithm during the optimization step. A larger number of optimization steps allows local search to design slightly better schedules, but this comes at the cost of increased compute time. Figure 2 presents a diagram describing the local search process.

In this study, we concentrate on a randomly-generated 10-person test population. Each of the 10 people in the test population is allowed to create schedules from an activity set comprised of two activities. The first activity is allowed to be between 1 and 24 hours long (allowing for a variety of short- or long-duration activities, such as personal care, shopping, and medical appointments). The second activity is set to be between 4 hours and 10 hours (forcing longer-duration activities, such as work, home, and sleep). The weekend factor for both activities is 1.0 (indicating that the activities are equally likely to occur during the weekend as they are during the week). The maximum travel time for each activity is fixed at 2 hours. Activities are allowed to start and end at any point during the day.

### 2.2 Sample entropy

Certain human activities occur with a high degree of regularity (e.g., working, going home), while others occur more erratically (e.g., medical treatment, social recreation) (Bhat et al. 2004; Kitamura and Hoorn 1987; Kitamura et al. 2006; Schlich and Axhausen 2003). Here, we develop a procedure to choose DASim parameters (α_{*u, p*}_, β_{*u, p*}_, γ_{*u, p*}_, *C*, *D*) that ensure spontaneity or regularity in an activity. We use the *sample entropy* (SampEn) statistic to detect regularity in a time series associated with a schedule.

SampEn was first introduced by Richman and Moorman (Richman et al. 2004; Richman and Moorman 2000) in response to Pincus’ seminal work on *approximate entropy* (ApEn) (Pincus 1991). Entropy quantifies the amount of order or disorder in a system. Ordered systems yield low entropy while disordered or chaotic systems yield high entropy. For a time series, this usually means that a low entropy system will have repeated changes or will remain constant, while a high entropy time series will have unpredictable changes that are highly variable. ApEn was originally developed to analyze regularity in medical and biological time series, specifically neonatal heart rates. It is still commonly used in medical literature (Goldberger et al. 2002; Hornero et al. 2005, 2006; Pincus and Goldberger 1994; Varela et al. 2003) and has also been applied to a variety of other fields including finance (Pincus and Kalman 2004) and human factors engineering (McKinley et al. 2011). SampEn improves on ApEn in several ways; most notably, it is a less biased statistic and requires about half the computing time (Richman and Moorman 2000).

SampEn computes the conditional probability that if a finite time series repeats itself within a tolerance *r* for *m* points, then it will also repeat itself for *m* + 1 points, without allowing self-matches (Lake et al. 2002). Small values of SampEn (values close to zero) indicate signal regularity (i.e., an ordered system), while relatively larger values indicate less regularity (i.e., a more disordered system). SampEn is still a comparative measure; there is no single threshold above which we may say that any arbitrary signal is *irregular*. It must be judged relative to the problem being addressed.

In our simulations, SampEn is used to quantify regularity of demand hours for activities on an hourly basis (i.e., *m* = 1). It is common practice to set *r* equal to some fraction of the standard deviation (σ) of the data being analyzed, allowing measurements on datasets with different amplitudes (Richman and Moorman 2000); thus, we set *r* = 0.2σ, where σ is computed from DASim’s demand hours output. We use the SampEn implementation written in C provided by PhysioNet.^3^

### 2.3 Global sensitivity analysis

**Fig. 3:**
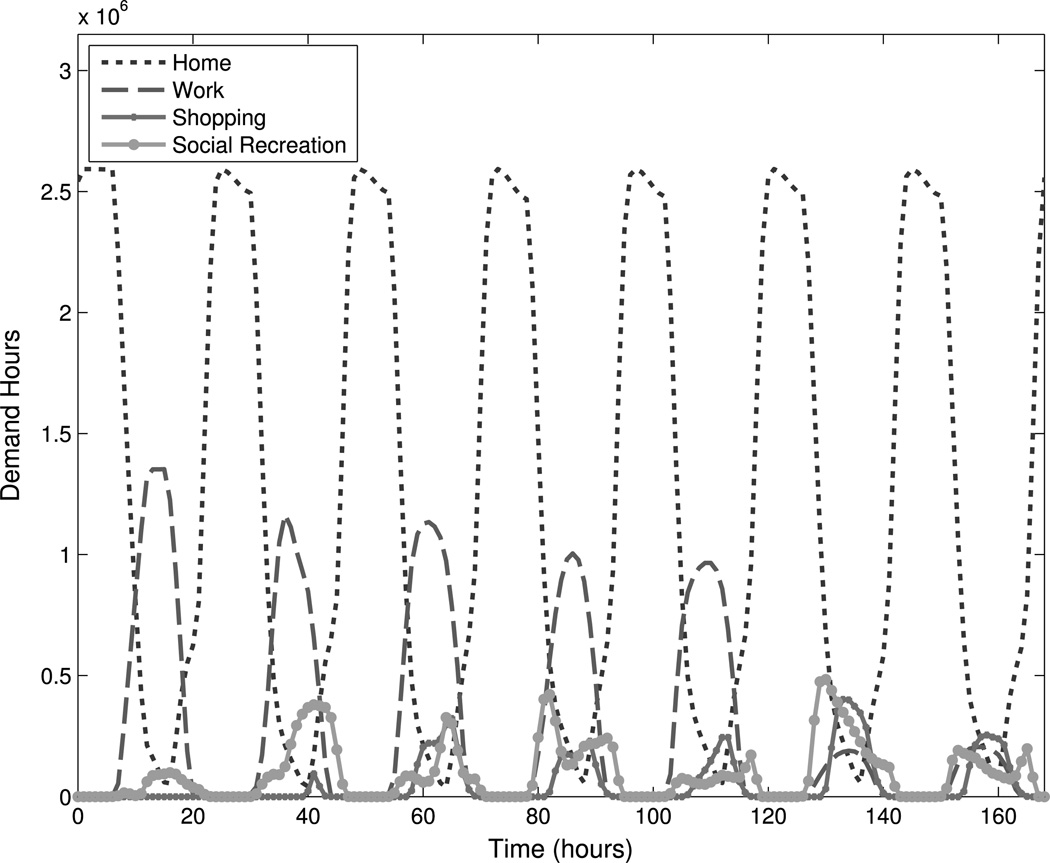
Sample DASim output. DASim outputs “demand hours” on an hourly basis. Demand hours represent the total number of people participating in an activity at a given time. This example shows output for four activities over the course of two weeks for the Minneapolis-Saint Paul region in Minnesota. The DASim parameters used for this figure were selected by hand to agree with a desired schedule of activities (e.g., regularity, peaks and valleys of a certain size, etc.). Some activities (e.g., home and work) occur with obvious regularity while others (e.g., social recreation) occur more sporadically. We use the sample entropy statistic to quantify regularity

We perform a global sensitivity analysis on the SampEn values computed from 12-week DASim simulations with respect to the input parameters for the priority and utility functions. DASim outputs *demand hours* on an hourly basis for each activity, which represent the total number of people participating in an activity aggregated over one hour. For our 12-week simulation period, DASim outputs 2,016 demand hour data points. Figure 3 shows a one-week sample of DASim output (168 demand hour data points). Note how regularity is evident for home and work activities on a 24-hour cycle.

We label two activities, 𝒜_1_ and 𝒜_2_, for our 10-person population. For each activity, we define utility and priority functions as in (1) using parameter sets (α_*u*_1__, β_*u*_1__, γ_*u*_1__, α_*p*_1__, β_*p*_1__, γ_*p*_1__) for 𝒜_1_ and (α_*u*_2__, β_*u*_2__, γ_*u*_2__, α_*p*_2__, β_*p*_2__, γ_*p*_2__) for 𝒜_2_ along with global optimization parameters *C* and *D*. A SampEn value is computed for each activity from the DASim demand hours output. For brevity, we denote the set of inputs to a given schedule by:

(3) $$\theta =(C,D,\alpha_{u_{1}},\beta_{u_{1}},\gamma_{u_{1}},\alpha_{p_{1}},\beta_{p_{1}},\gamma_{p_{1}},\alpha_{u_{2}},\beta_{u_{2}},\gamma_{u_{2}},\alpha_{p_{2}},\beta_{p_{2}},\gamma_{p_{2}}).$$

The main notation used throughout the sensitivity analysis is as follows: we will refer to each of the variables in a given θ ∈ ℝ^14^ using subscripts, θ_*j*_ for *j* = 1, 2, 3, …, 14. Note that we will also be taking multiple samples of θ parameter sets to construct a statistical model of the SampEn. From *M* samples of θ parameter sets, we form the *M* × 14 sample matrix Θ whose rows are the samples of the θ parameter sets. We will then use the notation Θ_*i, j*_ to refer to the *j*^th^ parameter in the *i*^th^ sample with *i* = 1, 2, …, *M* and *j* = 1, 2, …, 14. A single subscript will refer to a row of Θ, so Θ_*i*_ is the *i*^th^ sample parameter set, *i* = 1, 2, …, *M*.

The dynamic scheduling and SampEn computation define the function

$$\vec{\text{SampEn}}(\Theta )=({\text{SampEn}}_{1}(\Theta ),{\text{SampEn}}_{2}(\Theta )),$$

with entries corresponding to each activity. We calculate *Sobol-Saltelli sensitivity indices* (Oakley and O’Hagan 2004; Saltelli 2008) for SampEn_1_ (Θ) and SampEn_2_ (Θ); here, we explain this process for just one of these. We denote the scalar **Se**, for one activity, without index, as **Se** = SampEn_*n*_ (Θ) for *n* = 1 or 2 (this is done for brevity in the following formulas). First, we specify an allowable range for each of the parameters, $\theta_{j}\in [\theta_{j}^{-},\theta_{j}^{+}]$, and consider θ_*j*_ as a uniformly distributed random variable on $\left[\theta_{j}^{-},\theta_{j}^{+}\right]$. This makes the SampEn for each activity a random variable with variance determined by each of the ranges of θ_*j*_ and its dependence on each of these variables.

We compute *first order Sobol-Saltelli sensitivity indices*, defined as:

(4) $$S_{j}=\frac{V(𝔼(Se|\theta_{j}))}{V(Se)}\text{for}j=1,2,3,\ldots ,14,$$

where *V* (**Se**) denotes the variance and 𝔼(**Se**|θ_*j*_) denotes the expectation of the conditional random variable **Se**|θ_*j*_. In the variance of the conditional expectation, *V* (𝔼(**Se**|θ_*j*_)), the expectation integral is taken over all variables except θ_*j*_, with the *j*^th^ variable fixed, and the variance is an integral over just θ_*j*_. These sensitivity indices represent the fraction of the variance in **Se** that is attributed to variation in θ_*j*_. An equivalent interpretation of *S_j_* is the expected fraction by which the variance in **Se** will be reduced, if the value of θ_*j*_ is fixed.

The *S_j_* rank the importance of each variable, θ_*j*_, in terms of how much change in **Se** is present when θ_*j*_ is varied within $\left[\theta_{j}^{-},\theta_{j}^{+}\right]$. However, the first order indices do not provide a complete ranking of parameter importance when simultaneous variation in sets of variables is allowed (Homma and Saltelli 1996). To quantify importance of a parameter while accounting for its interaction with other variables, we calculate *total effect sensitivity indices*:

(5) $$S_{j}^{T}=\frac{V(Se)-V(𝔼(Se|\theta_{\sim j}))}{V(Se)}=1-\frac{V(𝔼(Se|\theta_{\sim j}))}{V(Se)}\;\theta_{\sim j}=(\theta_{1},\theta_{2},\ldots ,\theta_{j-1},\theta_{j+1},\ldots ,\theta_{14}).$$

The $S_{j}^{T}$ represent the expected fraction of the variance in **Se** remaining, if all parameters except θ_*j*_ are fixed. This then accounts for how the remaining variance due to θ_*j*_ can change, if θ_~*j*_ is fixed at different values.

To rank the importance of each variable with respect to the variation in **Se**, we examine the entire set (Saltelli 2008):

(6) $$\{S_{j},S_{j}^{T}:j=1,2,\ldots ,14\}.$$

The sensitivity indices have some desirable properties when applied to ranking parameters with respect to their influence on the variance of an output. If a variable does not influence the function at all, *S_j_* = 0, and if a variable does not have any interaction with the other variables, $S_{j}=S_{j}^{T}$ (Sobol 2001). In all situations, we have (Sobol 2001):

(7) $$0\leq S_{j}\leq S_{j}^{T}\leq 1.$$

Regardless of the utility of these sensitivity indices, they can be difficult to interpret since they are dependent on the distribution of the input parameters. Changing the interval for the parameter θ_*j*_, $\left[\theta_{j}^{-},\theta_{j}^{+}\right]$, changes the indices *S_j_* and $S_{j}^{T}$. Moreover, since this interval affects *V* (**Se**), changes to the interval θ_*j*_ may affect the sensitivity indices of other parameters. This is due to the global nature of the Sobol-Saltelli sensitivity indices and may cause interpretation difficulties due to parameter interdependencies.

A traditional Monte Carlo approach to compute the sensitivity indices is computationally expensive due to the repeated/iterated terms such as *V* (𝔼(**Se**|θ_*j*_)). A variety of approaches have been suggested to bring down the computational cost (Homma and Saltelli 1996; Marrel et al. 2009; Oakley and O’Hagan 2004; Saltelli 2002; Saltelli et al. 1999).We compute approximations to the sensitivity indices using a statistical surrogate model (Marrel et al. 2009; Neal 1997; Oakley and O’Hagan 2002, 2004), or emulator, for the function **Se** (θ). The emulator uses a Gaussian process regression (Higdon et al. 2008; Marrel et al. 2009; Neal 1997; Williams et al. 2006), which consists of fitting a Gaussian process **Se**_*g*_ (θ; η) to samples of **Se** (θ) taken at different θ parameter sets specified by the rows of the *M* sample matrix Θ.

The Gaussian process emulator (MacKay 1998; Neal 1997) is constructed using Bayesian Gaussian process regression. For a more complete description of this process we refer the reader to Higdon et al. (2008), Marrel et al. (2009), Oakley and O’Hagan (2002, 2004), Williams et al. (2006). First, the emulator **Se**_*g*_ (θ; η) is a stochastic process in the variable θ ∈ ℝ^14^ with state variable η. It has the property that the evaluation at any finite number of θ samples (**Se**_*g*_ (Θ_1_), **Se**_*g*_ (Θ_2_), …, **Se**_*g*_ (Θ_*M*_))^*T*^ is a Gaussian-distributed *M*-dimensional random vector, having mean μ = μ(Θ_1_, Θ_2_, …, Θ_*M*_) and covariance Cov = Cov (Θ_1_, Θ_2_, …, Θ_*M*_).

In the Bayesian regression approach, **Se**_*g*_ is constructed from samples of the output **Se**_*i*_ = **Se** (Θ_*i*_), *i* = 1, 2, …, *M*. The mean and covariance of **Se**_*g*_ are defined so that realizations of the simulated values have a maximized posterior probability given a prior distribution on the form of the covariance. The form for the covariance is specified so that when evaluating at a new parameter set, θ*, the variance of **Se**_*g*_ (θ*) increases for θ* further from the samples in the matrix Θ and goes to zero, if θ* lies in this sample set. The mean of **Se**_*g*_ (θ*) is related to the sampled values so that it is equal to **Se**_*i*_ for θ* = Θ_*i*_. Thus, **Se**_*g*_ (θ; η) is an interpolant of the sample values.

Sensitivity indices of 𝔼_η_ (**Se**_*g*_ (θ; η)) can be computed quickly once **Se**_*g*_ is constructed from a sample set. We refer to Marrel et al. (2009), Oakley and O’Hagan (2004) for this computation. To construct the Gaussian process and to compute the sensitivity indices, we used the Los Alamos GPM/SA code^4^ (Higdon et al. 2008; Williams et al. 2006).

### 2.4 Global optimization

Our goal is to find values for each of the parameters in θ for which SampEn, for the given activities, is either minimized (for increased regularity in scheduling) or maximized (for increased spontaneity). Optimizing over the complete 14-dimensional parameter space can be costly. Note that this 14-dimensional space is only for two activities; each additional activity adds 6 new parameters. Therefore, analyzing five activities would require optimization over a 32-dimensional space, which is computationally expensive for updating a schedule dynamically.

We use the global sensitivity indices to reduce the dimensionality of the optimization problem and identify parameters that contribute very little to the variance of SampEn. In an optimization step, these parameters are then fixed, and the remaining parameter space is searched using a global optimization procedure. If the number of parameters to which SampEn is sensitive is small, this can potentially result in a cheaper optimization procedure.

Schedules may be generated so that each activity has a desired level of regularity/irregularity by maximizing a single objective function, *J* (θ), involving the SampEn statistics for each activity in the schedule. We define the objective function for a schedule of *N* activities, 𝒜_1_, 𝒜_2_, …, 𝒜_*N*_, by

(8) $$J(\theta )=\sum_{i=1}^{N}w_{i}{\left|{\text{SampEn}}_{i}(\theta )-L_{i}\right|}^{2}.$$

Here, the desired levels of SampEn for each activity are denoted by *L_i_* and weights, *w_i_*, are associated with each activity to control the importance of each term in the maximum of *J* (θ). It is important to note that we include the square of the absolute value in our objective function so that *J* (θ) is smooth.

Maximization of these types of objective functions can attain specific goals allowing for more specificity in schedule design with regards to mixtures of regular and irregular activities. For instance, in a two-activity schedule we may choose *w*_1_ = *w*_2_ = 1 and *L*_1_ = *L*_2_ = 0 to obtain the objective function

(9) $$J(\theta )={\text{SampEn}}_{1}{\left(\theta \right)}^{2}+{\text{SampEn}}_{2}{\left(\theta \right)}^{2}.$$

Maximization of (9) generates schedules where both activities have a high SampEn and, therefore, have irregular activity demand hour time series for both activities. Alternatively, taking *w*_1_ = 1, *w*_2_ =−1, and *L*_1_ = *L*_2_ = 0, we get

(10) $$J(\theta )={\text{SampEn}}_{1}{\left(\theta \right)}^{2}-{\text{SampEn}}_{2}{\left(\theta \right)}^{2}.$$

Maximization of (10) will generate schedules in which 𝒜_1_ has highly irregular demand and 𝒜_2_ has very regular demand. More specific conditions can be met by specifying non-zero levels of SampEn for each activity. Setting *w*_1_ = −1, *L*_1_ = 0.9, *w*_2_ = −0.5, and *L*_2_ = 1.5 we get

(11) $$J(\theta )=-{\left|{\text{SampEn}}_{1}(\theta )-0.9\right|}^{2}-\frac{1}{2}{\left|{\text{SampEn}}_{2}(\theta )-1.5\right|}^{2}.$$

When maximizing (11) the contribution from the term involving SampEn_1_ (θ) has twice the effect of the contribution from the term involving SampEn_2_ (θ). Therefore schedules will be generated with SampEn_1_ (θ) ≈ 0.9, SampEn_2_ (θ) ≈ 1.5, and SampEn_2_ (θ) farther from 1.5 than SampEn_1_ (θ) is from 0.9.

We use the *harmony search* (HS) global optimization algorithm (Geem et al. 2001) to explore the parameter space. HS is a metaheuristic search algorithm, inspired from the improvisation process of jazz musicians, that optimizes (minimizes or maximizes) a certain objective function. Recently, HS has been successfully applied to a variety of problems including water distribution network design (Geem 2006b), parameter estimation (Kim et al. 2001), combined heat and power economic optimization (Vasebi et al. 2007), and even sudoku solving (Geem 2007). In many cases, it has been shown to outperform other commonly used search algorithms, such as simulated annealing (Kirkpatrick et al. 1983), tabu search (Glover 1989, 1990), and evolutionary algorithms (Bäck and Schwefel 1993).

In HS, sets of parameters (referred to as a *harmonies*) are randomly chosen (*improvised*) until the *harmony memory* is filled. A new harmony is improvised according to a set of rules: each parameter (*note*) may be chosen via *random selection* or *memory consideration* with an optional *pitch adjustment* (adjusting a parameter up or down slightly). The goodness of the new harmony is computed (in this case, the sum of the SampEn statistics for each activity), and if the harmony is better than the worst harmony stored in the harmony memory, the new harmony replaces the previously stored value.

HS features five main parameters: max_imp determines the maximum number of improvisations (iterations), hms is the harmony memory size (the number of best harmonies that should be remembered), hmcr is the harmony memory consideration rate (how often a note is chosen via memory consideration as opposed to random selection), par is the pitch adjusting rate (how often pitch adjustment is invoked), and mpap is the maximum pitch adjustment proportion (size of the perturbation).

A number of improvements and changes have been suggested since HS’ first introduction. One change added the notion of *ensemble consideration*, an operation that considers relationships between decision variables (Geem 2006a). Another modification, dubbed improved harmony search, dynamically modifies the par and mpap parameters as the search progresses (Mahdavi et al. 2007). Global-best harmony search removes the mpap parameter altogether by altering the pitch-adjustment step so that values are drawn from the best harmony in the harmony memory (Omran and Mahdavi 2008). Most recently, a parameter setting-free variation was introduced that dynamically modifies both hmcr and mpap as the search progresses (Geem and Sim 2010).

For this study, we implemented the original HS algorithm in Python. The source code has been open-sourced and is available on GitHub.^5^ At the start of our HS optimization for DASim, *C*, *D*, β_*u*_, and β_*p*_ are allowed to vary in the range [0, 1], while α_*u*_ and α_*p*_ are allowed to vary in the range [0, 86400]. The parameters γ_*u*_ and γ_*p*_ are allowed to vary in the range [0, 10]. Notice, there is no need to normalize all inputs to a common range since the sensitivity indices rank the inputs relative to their ranges. HS is then combined with global sensitivity analysis to reduce the dimensionality of the search space, which is done iteratively as follows:

#### Harmony Search with Global Sensitivity Analysis Algorithm

**Fig. 4:**
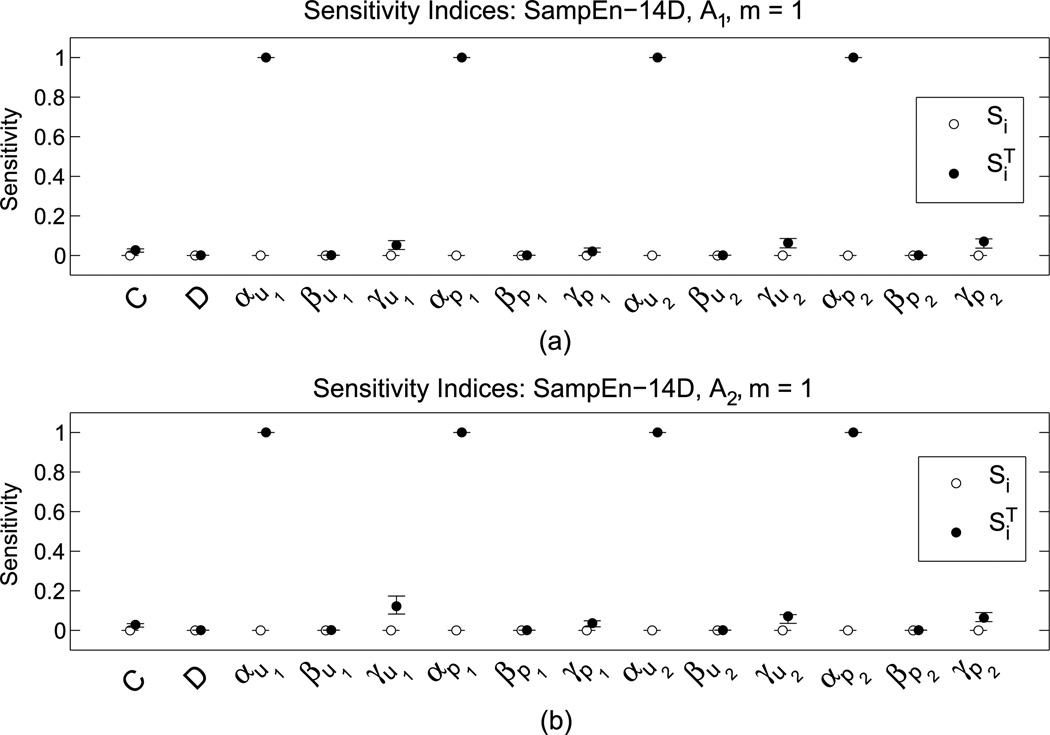
Sensitivity indices for the sample entropy of time demands for two different activities on an hourly basis (i.e., when *m* = 1). *Closed circles* are the *total effect sensitivity indices*, $S_{i}^{T}$, and open circles are the *first order sensitivity indices*, *S_i_*. For each sensitivity index, the subindices on *S_i_* and $S_{i}^{T}$ indicate the corresponding variable labeled on the *x*-axis. Plots (**a**) and (**b**) are sensitivity indices corresponding to activity 1 and 2, respectively. In both (**a**) and (**b**), we see that the α-parameters are much more sensitive than the other parameters. This implies that variations in α-parameters account for the majority of the variation in sample entropy values. One can also see some contribution to the variance by the γ -parameters. Moreover, since the first order sensitivity indices are near zero and the total effect indices are near one, we can tell that the interaction of the α-parameters is very nonlinear. In practice this means that a schedule’s regularity is most sensitive to the location of the utility and priority function and less sensitive to its shape

1. Provide allowable intervals for each parameter $\theta_{j}\in [\theta_{j}^{-},\theta_{j}^{+}]$, *j* = 1, 2, …, 14.
2. *M* samples of SampEn are taken at different parameter sets in the sample matrix Θ_*M* × 14_.
3. Samples used to construct a Gaussian process emulator, **Se**_*g*_ (θ; η).
4. Sensitivity indices, $\left\{S_{j},S_{j}^{T}\right\}$, *j* = 1, 2, …, 14, are computed from **Se**_*g*_ (θ; η).
5. A subset of parameters, (θ_*k*_1__, θ_*k*_2__, …, θ_*k_d_*_), with high sensitivity values (see Fig. 4) are chosen on which to perform HS. The remaining parameters are fixed (note that we arbitrarily fix them at the mean value of their interval). Here, we use notation for an arbitrary subset of distinct parameter subscripts of size *d* ≤ 14, {*k*_1_, *k*_2_, …, *k_d_*} ⊂ {1, 2, …, 14}.
6. HS is performed over the parameter subset to maximize a given functional of SampEn statistics for each activity.

Each 12-week simulation of DASim for the 10-person test population takes approximately 5 seconds wall time to complete. We initialize max_imp to 2000, hms to 50, hmcr to 0.75, par to 0.5, and mpap to 0.25. HS consistently converged to solutions of approximately the same fitness over many test runs, each with initial harmonies selected uniformly at random. As a result, we determined that a parameter sweep of the HS parameters was unnecessary.

## 3 Results

The global sensitivity analysis (Fig. 4) shows that the offset parameters, (α_*u*1_, α_*p*1_, α_*u*2_, α_*p*2_), have the largest effect on the variation of the sample entropy for both activities. Thus, these α parameters have the most impact on regularity. Recall that, as seen in Table 2, α_*u*_ and α_*p*_ control the activity duration in the utility and activity frequency in the priority, respectively.

**Fig. 5:**
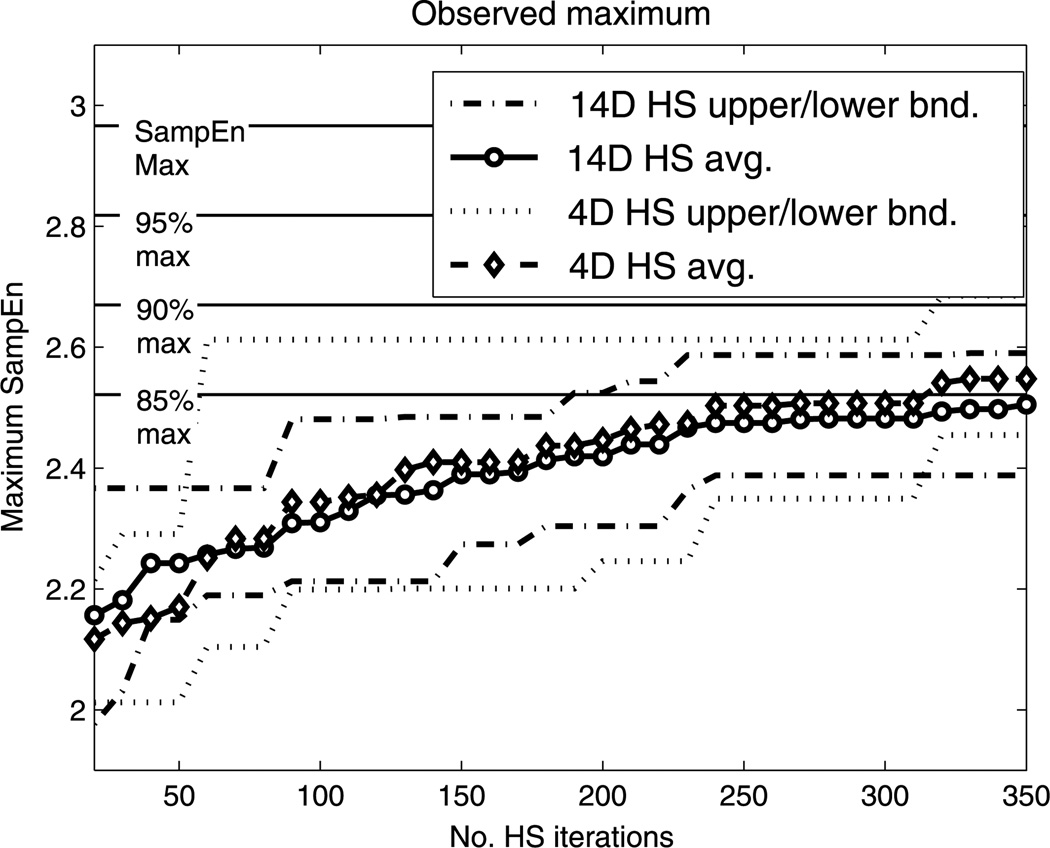
Comparison of sum of SampEn maximization strategies. Here, we are showing the convergence of HS to the maximum sum of SampEn values, thus maximizing the SampEn of both activities simultaneously. The searches are over the entire 14-dimensional space and over the four-dimensional subspace of the sensitive α-parameters. We see that the search over the small subspace performs better on average for a number of HS iterations under 350. Moreover, the small subspace search has a significant chance to achieve within 85 % of the maximum SampEn in under 200 iterations

We adjusted schedules to consist of two irregularly performed activities. This was done by maximizing (9), adjusting only four parameters, (α_*u*1_, α_*p*1_, α_*u*2_, α_*p*2_), using the HS global optimization algorithm. The results of the four-parameter space were compared against tuning the entire 14-dimensional space. In Fig. 5, we show that, for small numbers of HS iterations (i.e., less than 350), the four-dimensional subspace search performs better on average; we can reach much closer to the maximum SampEn in fewer iterations than a search over the whole parameter space. While running 500 iterations of HS over the whole parameter space will result in a better maximum SampEn, our results show that HS over the four-dimensional space will reach 90 % of the maximum SampEn with fewer than 100 iterations. Therefore, the search space should be chosen based on computational requirements.

We performed random sampling over the entire 14-dimensional parameter space and compared the variance in the SampEn for each activity against only varying the α-parameters. Our results show that the variation in SampEn caused by only varying the α-parameters was responsible for about 99 % of the variance in SampEn when the entire parameter set was allowed to vary. This result was consistent for each activity. This shows that our sensitivity analysis with the emulation gives realistic results and that optimization over the four-dimensional parameter space will suffice to approximate the minimum or maximum of the sample entropy or a functional thereof.

**Fig. 6:**
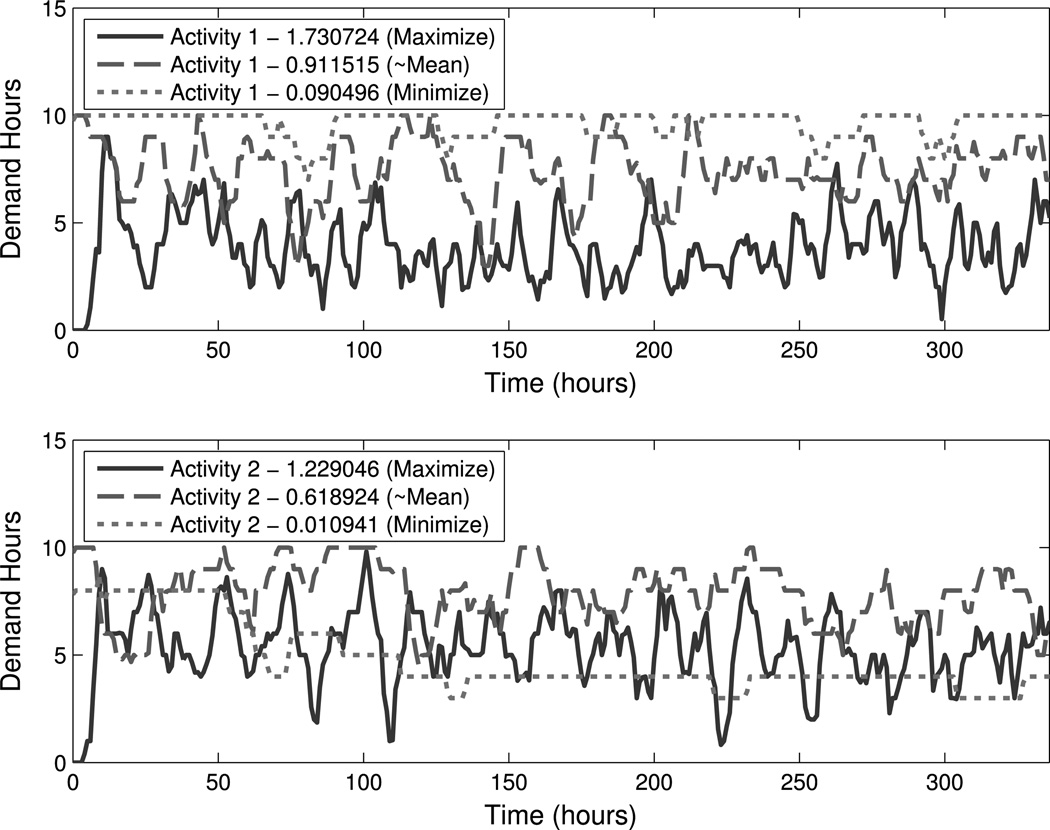
Demand hours resulting from SampEn optimization. These plots show demand hour output for the two test activities, denoted 𝒜_**1**_ and 𝒜_**2**_, over the course of two weeks for the Minneapolis-Saint Paul region in Minnesota for the 10-person population. The *top plot* shows demand hours for 𝒜_**1**_, and the *bottom plot* shows demand hours for 𝒜_**2**_. The parameters for DASim were chosen using HS, which was directed to either maximize or minimize SampEn preferentially for one activity with *m* = 1. HS coupled with sensitivity analyses allow us to design order and disorder into DASim after first reducing the parameter search space. In both plots, as SampEn decreases, demand hour variability and range visibly reduces. Note that for activities considered on an hourly basis (i.e., when *m* = 1), the most regular time series (i.e., minimal SampEn) would be a constant line while the most chaotic time series (i.e., maximal SampEn) would be far from constant. We can see that, for both activities, as SampEn increases, regions of constant demand hours become more rare

The maximization of SampEn over the α-parameters creates a schedule with a great deal of spontaneity. In addition to maximizing the sum of both SampEn statistics, we preferentially maximized and minimized each activity individually, ignoring the other activity. These SampEn-minimized and -maximized schedules, along with a schedule that has SampEn equal to the mean of the minimized and maximized Samp-En schedules, are shown in Fig. 6. We see a visual difference: DASim output for a maximized SampEn schedule is more variable over a larger range than minimized SampEn schedules. Also, when SampEn is minimized, regions of constant demand hours are more prevalent, which is to be expected for activities considered on an hourly cycle (i.e., when *m* = 1).

## 4 Discussion

This study focuses on schedule realism in a human activity model, but the methods presented here are generic and can be applied to a variety of other problems where a specific property in a high-dimensional model is desired. These types of high-dimensional tuning/optimization problems are ubiquitous in modern complex computer simulations. Thus, there is a significant need for methods of automatic tuning that incorporate systematic dimension reduction. Our combination of global sensitivity analysis and a global optimization method is effective for the application presented here. Additionally, it is sufficiently general to warrant application in many other areas.

Dynamic scheduling for synthetic populations is necessary to make simulations of human behavior phenomena more realistic. The dynamic scheduling program DASim was designed to aid in large-scale agent-based infrastructure simulation (e.g., transportation and epidemic modeling). DASim can generate schedules that are different over demographics and change in response to events, such as disease outbreaks and non-pharmaceutical interventions.

To evaluate the realism of a dynamically-generated schedule, we must select metrics on which it should be evaluated. We presented a method for tuning a dynamic scheduling model for schedule regularity, which we quantify using the sample entropy (SampEn) statistic applied to population demand hours. Adjusting the SampEn statistic requires working with a high-dimensional optimization problem. We used global sensitivity analysis and statistical surrogate models to significantly lower the dimensionality of the search space. A global optimization algorithm, harmony search (HS), was used to efficiently tune the degree of regularity of a schedule.

Some of the major results of our study include:

– Demand hour regularity of activities over a population can be controlled by tuning the SampEn statistic.
– DASim parameters that most influence the SampEn statistic can be identified using global sensitivity analysis combined with a statistical surrogate model. We determined that the α parameters in the utility and priority functions have the largest effect on the variation of the sample entropy of an activity.
– DASim parameters that result in close to optimal (i.e., minimized/maximized) SampEn values can be discovered using HS. Furthermore, this can be done efficiently with many fewer iterations by searching a parameter subspace determined by global sensitivity analysis first (just the α parameters in this study).

While we have shown how to reduce the search space and computation time when analyzing parameter importance under a particular metric, this process still takes a significant amount of compute time. We tuned our parameters in a reduced problem environment, using a 10-person population. Although this approach works for the measure of regularity discussed in this paper, it may not work for more complex measures of interest. Our initial search space of 14 dimensions is still relatively small; some simulations may have many tens, hundreds, or even thousands of dimensions. Understanding parameter importance and interactions in such high-dimensional spaces may prove difficult or even impossible in some instances using our methods.

Our analysis is based on hourly regularity for demand hours of schedules. Many other granularities may be desirable. For example, work may be regular every 12 hours. Some studies suggest that the size of the dataset be at least 10^*m*^ and preferably at least 30^*m*^ in the approximate entropy (ApEn) algorithm (Pincus and Goldberger 1994). While this is certainly possible for small values of *m* (recall that *m* = 1 in this paper), larger values of *m* quickly become problematic (e.g., work would require at least 10^12^ demand hour data points). Alternative measures of regularity may be considered for larger values of *m*.

We are considering other evaluative measures to quantify additional properties, beyond regularity, of a schedule’s realism. Here, we analyzed measures of regularity of time usage and found that it is controlled by a small set of the defining parameters in the model. Another possibility would be to look at quantifying the efficiency of a dynamically-generated schedule in terms of location usage, whether an individual’s schedule is geographically arranged in a sensible way given his or her current location. One could also look at the total percentage of time spent on an activity. Evaluation of a measure of each of these effects would lend testable realism to a generated schedule for a population. The use of statistical emulation, global sensitivity analysis, and optimization as demonstrated here would then allow for efficient tuning of these measures.

For models that rely on human activity patterns and movement, such as disease and infrastructure models, capturing realistic activity patterns is crucial for decision support. Therefore, new techniques such as the ones proposed here are needed for analyzing high-dimensional problems. However, more research still needs to be done related to efficiently solving these problems computationally and understanding human activity patterns and behavior.

## Figures and Tables

**Table 1:** Example activity set. Each demographic group has a different set of activities, its own utility and priority functions, time and distance constraints, and weekend factor

Group	Home	Work	Shop	Leisure	Medical	Day Care	School
Child (0–5)	✓			✓	✓	✓	
Youth (6–18)	✓			✓	✓		✓
Worker (19–64)	✓	✓	✓	✓	✓		
Senior (65+)	✓		✓	✓	✓		

**Table 2:** Parameters used in DASim’s utility and priority functions. Each parameter in DASim is shown with a brief description of what it influences and the ranges over which will allow it to vary in our optimization. Figure 1 shows how varying these parameters changes the sigmoid function in practice. Refer to Joh et al. (2001) for more details on these parameters

Parameter	Description	Range
α_{*u,p*}_	The α parameter determines the location of the sigmoid function along the X-axis. In the case of utility, larger α values result in longer-duration activities. Larger α parameters in priority functions result in less frequent activities.	[0, 86400]
β_{*u,p*}_	The β parameter determines the slope of the sigmoid function. For utility functions, larger β values limit the range of desirable activity durations. For priority functions, larger β values increase how rapidly an activity becomes important once enough time has elapsed since last execution.	[0, 1]
γ_{*u,p*}_	The γ parameter determines the inflection point in the curve. It defines how quickly the sigmoid’s concave region transitions to the convex region. For utility functions, larger γ values result in a more drastic concave to convex transformation, which in turn reduces activity duration range. Larger γ values in priority functions result in a larger range of time between activities.	[0, 10]

**Table 3:** Operators used by DASim when designing schedules. The local search metaheuristic randomly selects operators in order to create a new valid schedule from the current schedule. The objective function then compares the current schedule against this new schedule, as described in Fig. 2

Operator	Description
Delete-IncreaseDuration	Delete an activity and increase the durations of the activities surrounding the deleted activity.
AdjustDuration	Change the duration of two consecutive activities.
Substitute	Replace an existing activity with another.
DecreaseDuration-Insert	Decrease the duration of two consecutive activities and insert a new activity.
Append	Add a new activity.

## Footnotes

1 http://www.census.gov/.

2 http://www.dnb.com/.

3 http://www.physionet.org/physiotools/sampen/.

4 http://www.stat.lanl.gov/source/orgs/ccs/ccs6/gpmsa/gpmsa.html.

5 https://github.com/gfairchild/pyHarmonySearch.
